# Amodal Segmentation and Trait Extraction of On-Branch Soybean Pods with a Synthetic Dual-Mask Dataset

**DOI:** 10.3390/s25206486

**Published:** 2025-10-21

**Authors:** Kaiwen Jiang, Wei Guo, Wenli Zhang

**Affiliations:** 1 School of Information Science and Technology, Beijing University of Technology, Beijing 100124, China; kecin@emails.bjut.edu.cn; 2 Graduate School of Agricultural and Life Sciences, The University of Tokyo, Tokyo 113-8657, Japan

**Keywords:** soybean, pod phenotyping, amodal instance segmentation, synthetic data, occlusion reasoning, Swin Transformer, seeds per pod, morphology-based analysis

## Abstract

We address the challenge that occlusions in on-branch soybean images impede accurate pod-level phenotyping. We propose a lab on-branch pipeline that couples a prior-guided synthetic data generator (producing synchronized visible and amodal labels) with an amodal instance segmentation framework based on an improved Swin Transformer backbone with a Simple Attention Module (SimAM) and dual heads, trained via three-stage transfer (synthetic excised → synthetic on-branch → few-shot real). Guided by complete (amodal) masks, a morphology-driven module performs pose normalization, axial geometric modeling, multi-scale fused density mapping, marker-controlled watershed, and topological consistency refinement to extract seed per pod (SPP) and geometric traits. On real on-branch data, the model attains Visible Average Precision (AP) 50/75 of 91.6/77.6 and amodal AP50/75 of 90.1/74.7, and incorporating synthetic data yields consistent gains across models, indicating effective occlusion reasoning. On excised pod tests, SPP achieves a mean absolute error (MAE) of 0.07 and a root mean square error (RMSE) of 0.26; pod length/width achieves an MAE of 2.87/3.18 px with high agreement (R^2^ up to 0.94). Overall, the co-designed data–model–task pipeline recovers complete pod geometry under heavy occlusion and enables non-destructive, high-precision, and low-annotation-cost extraction of key traits, providing a practical basis for standardized laboratory phenotyping and downstream breeding applications.

## 1. Introduction

### 1.1. Background

As a globally important food and cash crop [1], soybean pod-level phenotypic parameters—including pod number, seeds per pod (SPP), and pod size [2]—are key indicators for evaluating yield components and breeding potential [3,4]. Efficient, accurate acquisition of these parameters (especially those derived from complete masks) is indispensable for understanding plant architecture, advancing smart soybean breeding, and enabling precise yield assessment [5]. In recent years, deep learning has driven image-based soybean pod phenotyping from manual measurement to automated processing [6,7,8], with related studies leveraging deep learning models [9] to parse pod images automatically and overcome the efficiency/accuracy limitations of traditional manual seed testing [10]. Among these traits, SPP directly reflects pod set and is critical to both yield components and cultivar evaluation; however, in on-branch scenes with common occlusion, accurate counting depends heavily on reliably recovering complete morphological information.

Soybean pod phenotyping typically occurs in two settings: field and laboratory. Field phenotyping focuses on whole-plant, whole-season dynamic monitoring of naturally growing plants to support yield prediction and field management optimization [11], but its variable environment (illumination, background, occlusion) [12] hinders high-precision automated measurement [13,14] of individual pod-level parameters. By contrast, laboratory phenotyping transfers harvested or stage-specific branch-bearing samples to a controlled environment (e.g., black backdrop, fixed illumination) for fine-grained measurements [15], enabling high-precision, automated acquisition of key pod geometric parameters and providing a reliable data basis for cultivar screening and genetic analysis [16,17]. This study targets laboratory on-branch phenotyping, emphasizing non-destructive SPP counting.

Even under controlled laboratory conditions, dense pod distributions and mutual occlusions remain pronounced. Existing deep learning instance segmentation methods (e.g., Mask R-CNN) primarily segment visible regions and do not reconstruct occluded parts—i.e., they do not generate amodal masks [18,19]. The absence of complete masks introduces systematic errors in estimating geometric quantities (e.g., pod length/width) and causes missed/merged counts in SPP estimation, ultimately constraining precise laboratory phenotyping.

### 1.2. Problem Statement and Scope

While deep learning-based soybean pod phenotyping methods achieve high accuracy in simplified laboratory settings with excised pods, they rely on labor-intensive manual excision and discard in situ spatial context—making them ill-suited for high-throughput, in-place phenotyping. Developing techniques for automated analysis of branch-retained samples is, therefore, a natural trend [20,21].

Unlike field studies focusing on whole-plant growth cycle monitoring [11], this study targets the laboratory on-branch scenario. Controlled imaging conditions (fixed illumination, black background) enable high-precision measurement of pod-level geometric traits (pod length, width, SPP) for cultivar screening, genetic analysis, and precise yield component assessment [16,17]. Even so, dense pod distributions and severe mutual occlusions remain pervasive, leaving two core challenges for efficiently obtaining accurate amodal masks (reflecting full pod geometry).

#### 1.2.1. Scarcity and Cost of High-Quality Annotations

Severe on-branch occlusion means human annotators typically only provide visible masks; amodal mask annotation for occluded regions is subjective, time-consuming, labor-intensive, and error-prone, with poor consistency. Existing synthetic data approaches [22] alleviate data scarcity but target excised pod settings (producing bounding boxes or visible masks). Pipelines tailored to on-branch, heavily occluded laboratory images—capable of generating high-quality visible and amodal labels simultaneously—are lacking. Constructing a high-quality dual-mask synthetic dataset for on-branch pods (reducing reliance on scarce real images and costly manual annotation) is thus the first key problem.

#### 1.2.2. Difficulty of Complete Mask Recovery Under Complex Occlusions

On-branch pods are densely packed with complex spatial layouts and frequent occlusion by branches, leaves, and neighboring pods—leaving parts of many pods invisible. Visible mask instance segmentation frameworks only predict exposed regions and cannot reconstruct occluded shapes; such partial masks induce systematic bias in complete geometric trait estimation (e.g., full pod length/width). Breaking these limitations to perceive/reconstruct occluded regions and obtain accurate amodal masks is the second key problem.

Guided by these challenges, we adopt a pipeline of “synthetic data → amodal segmentation → counting & geometry extraction” enabling stable, unbiased SPP estimation under occlusion.

### 1.3. Contributions

To address the two core issues (scarce dual-mask annotations and difficult complete-mask recovery under occlusion), this study proposes an amodal segmentation-based method for soybean pod phenotypic parameter extraction in laboratory on-branch settings. The method overcomes dense occlusions to recover complete pod geometry and provides a stable, automated solution for SPP counting. The key contributions are as follows.

#### 1.3.1. Synthetic Data Construction: Automatic Branch Pod Composition with Dual-Mask Labels

A prior-guided automatic composition/synthesis pipeline is proposed, defining pod poses, spatial arrangement rules, and occlusion patterns. It leverages pre-annotated, morphologically complete branch components and pod instances to automatically synthesize large numbers of realistic images (excised pod and on-branch) against a black background. Critically, the pipeline generates synchronized, precise visible and amodal masks for each pod. By exploiting prior knowledge of complete components, it produces amodal labels (otherwise difficult to annotate), substantially reducing dependence on real images and costly manual labeling while providing abundant, precisely labeled dual-mask training data.

#### 1.3.2. Amodal Segmentation: Improved Backbone with Coordinated Transfer Learning

An amodal segmentation framework is designed, integrating an improved Swin Transformer backbone [23] and a dual-head architecture [24] to predict both visible and amodal masks. A multi-stage transfer learning strategy progressively trains/fine-tunes the model on (i) synthetic excised pod data, (ii) synthetic on-branch data, and (iii) a small amount of real data—enabling accurate localization and full-shape recovery for densely occluded on-branch pods at low annotation cost and ensuring reliable downstream phenotypic extraction.

#### 1.3.3. SPP Counting: Amodal Mask-Guided Extraction

Under full-shape constraints from amodal masks, a structured counting pipeline is devised: pose normalization → axial geometric modeling → multi-scale fused density mapping → peak detection and watershed segmentation → topological-consistency refinement. This pipeline suppresses missed/merged counts caused by occlusion/adhesion, yielding stable, truth-conforming results. Combined with automatic acquisition of geometric indices (pod length, width, area), it supports high-throughput, non-destructive analysis of yield components.

The subsequent sections are organized as follows: Section 2 reviews related work across excised/lab/field settings and the amodal/synthetic literature; Section 3 details the materials and methods (synthetic data construction, amodal segmentation model, and SPP counting pipeline); and Section 4 presents the experimental results and analysis to verify the method’s effectiveness.

## 2. Related Work

This section reviews soybean pod phenotyping across the excised laboratory, the on-branch laboratory, and field settings, emphasizing SPP under occlusion and the need for amodal (complete) masks for unbiased geometry and counting.

### 2.1. Laboratory Studies on Excised Pods (Simplified Setting)

Excised pod studies remove pods from plants and image them against uniform, standardized backgrounds [25]. This markedly reduces background complexity, target overlap, and pose variation, thereby facilitating training of object detection and instance segmentation models with high accuracy [26,27]. However, excision is labor-intensive and costly, and it destroys in situ spatial information of pods on plants. As a result, such pipelines cannot faithfully reflect growth status or whole-plant yield composition and are less suitable for high-throughput phenotyping beyond controlled conditions.

Representative works span traditional and deep learning approaches. Uzal et al. [6] combined hand-crafted morphology/texture features with SVMs/CNNs to estimate SPP on single-pod images. Li et al. [28] proposed a point-based detection framework to localize seed positions and count seeds via a two-column CNN (TCNN), while Li et al. [29] developed SPM-IS (instance segmentation with Feature Pyramid Network and PCA) to extract traits (e.g., pod length) from excised samples. He et al. [25] improved YOLOv5 by embedding Coordinate Attention (CA) to enhance small-object features and paired it with a BP neural network to predict single-pod weight. To reduce excision and manual labeling burdens, Yang et al. [30] generated synthetic images from a small labeled set to build large mask-annotated datasets and simulate overlap for better segmentation performance. Despite these advances, all the above methods rely on pod excision and discard the on-plant spatial context, motivating the need for in situ, on-branch analysis techniques.

### 2.2. Laboratory Studies on On-Branch Pods (Complex Setting)

On-branch analysis preserves in situ spatial information (beneficial for understanding plant architecture and whole-plant yield potential [31]) but faces small targets, pose variability, and severe occlusion with stems, leaves, and neighboring pods. Research has followed three directions, each struggling to recover complete shapes under heavy occlusion, which limits accurate full-geometry estimation.

#### 2.2.1. Anchor-Based Object Detection

Detectors such as the YOLO family and Faster R-CNN [26,32] are widely used for on-branch pod detection/counting due to efficiency and strong localization. Zhou et al. [17] proposed the SPP extractor, improving YOLOv5s with Squeeze and Excitation (SE) attention to focus on occluded pods/stems and extract plant-level traits (e.g., plant height). Xiang et al. [33] built YOLO-POD on YOLOX, adding a pod count head and CBAM attention while replacing SPP with SPPF for lower memory usage. However, these methods only output bounding boxes and class confidences; they are insufficient for precise geometric traits (shape/size).

#### 2.2.2. Point-Based Object Detection

Point-based methods use points as the minimal prediction unit, localizing keypoints inside pods (often individual seeds) and grouping them to host pods via structural modeling or clustering. He et al. [34] proposed DEKR-SPrior, treating pods as “bodies” and seeds as “joints” (borrowing from human pose estimation) and introducing a structural prior (SPrior) to enhance inter-seed discrimination. With subgraph cropping and dynamic registration, it improves recognition in densely occluded regions, but outputs only seed points and their affiliations—limiting applicability for geometric morphometrics.

#### 2.2.3. Instance Segmentation with Visible Masks

Frameworks such as Mask R-CNN and SOLOv2 [27,35] generate visible masks for detected pods, enabling partial geometric measurements (e.g., visible area). However, heavy inter-pod occlusion is a core challenge; these methods do not perceive or reconstruct occluded regions [18]. High-quality training data are also scarce—amodal mask annotation (including occluded parts [19]) is difficult and labor-intensive, so available labels are limited to visible masks. Even well-trained models only predict visible regions, leading to mask fragmentation/missing regions that degrade phenotypic estimate accuracy. Yang et al. [30] used synthetic excised pod data for transfer learning to improve visible-part segmentation, but most synthetic efforts target excised pod settings; pipelines for automatically generating high-quality amodal labels for on-branch laboratory scenes remain lacking.

### 2.3. Field Studies on On-Branch Pods (Open Setting)

Field studies aim at non-destructive whole-plant or population-level recognition/counting under natural growth (supporting yield prediction and high-throughput breeding), but illumination variation, complex backgrounds (soil, weeds), and diverse poses often produce blurry boundaries and incomplete structures. At the macro level, Zhou et al. [36] proposed the Transformer-based SoybeanNet and the fused Swin Transformer with UNet for UAV-based pod counting; Mathew et al. [37] combined a ground depth camera with YOLOv7 for stable whole-plant counting. At the micro level, P2PNet-Soy [38] performs point-level detection with unsupervised clustering, and PodNet [39] employs YOLOv8-based segmentation with hierarchical feature aggregation. Overall, field methods emphasize counting and generally lack robust complete-shape reconstruction; high-precision geometry and SPP measurement thus remain more suitable for laboratory conditions.

### 2.4. Summary and Gaps

Across settings, detection methods offer coarse boxes that are insufficient for fine-grained phenotyping; point-level counting is vulnerable to merges/mis-assignment in dense occlusion; and visible mask segmentation cannot recover complete shapes. Compounded by the scarcity of dual-mask (visible + amodal) annotations, progress has reached a bottleneck. Consequently, obtaining unbiased, robust SPP counts and trustworthy geometric traits in heavily occluded on-branch scenes requires amodal masks and supervision regimes that expose models to controllable occlusions—motivating the dual-mask, synthesis-aided approach developed in this study.

## 3. Materials and Methods

**Figure 1:**
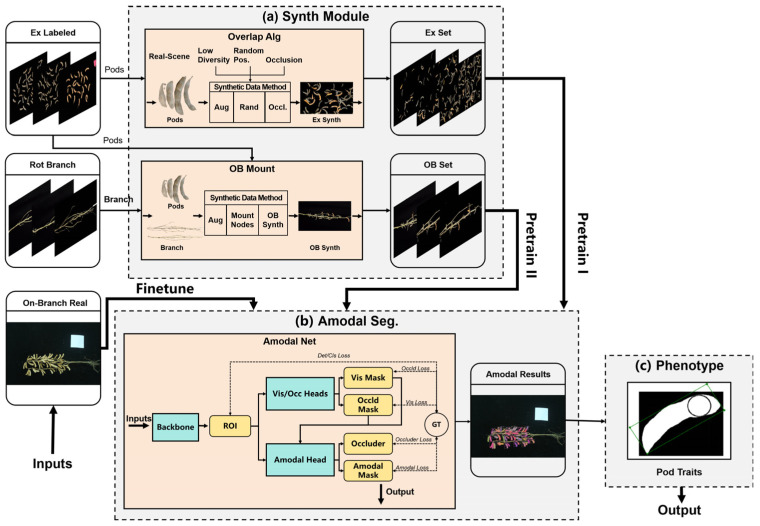
Schematic of the proposed low-annotation-cost phenotyping method based on synthetic data and amodal segmentation. (**a**) Synthetic dataset generation with dual-mask labels (visible + amodal); (**b**) amodal segmentation with dual heads and three-stage transfer learning; (**c**) phenotypic extraction from complete masks (length, width, area, SPP).

To address two coupled challenges in laboratory on-branch phenotyping—(i) the difficulty in obtaining complete masks under mutual occlusion and (ii) the scarcity/cost of high-quality annotations—we design a three-module framework that integrates synthetic data generation, amodal segmentation, and morphology-aware analysis for low-annotation-cost yet accurate phenotypic extraction. As shown in Figure 1, the framework comprises three modules.

The synthetic dataset generation module copes with incomplete samples and annotation difficulty caused by inter-pod occlusions. It includes two sub-pipelines—excised pod overlap simulation and on-branch mounting simulation—to produce two complementary datasets.

- The excised pod synthetic dataset comprises single-pod instances arranged on a black background at preset overlap ratios (10%, 30%, 50%) to learn fundamental pod morphology.
- The on-branch synthetic dataset includes excised pods automatically mounted onto branch structures (guided by priors like node distribution and occlusion patterns) to emulate realistic, complex on-branch scenes.

Both datasets synchronously provide visible and amodal masks (dual-mask labels) for each instance, furnishing precise supervision for downstream learning.

The soybean pod amodal segmentation module obtains complete masks of mutually occluded pods for accurate trait computation. The network employs visible region heads to predict exposed and occluded areas, plus an amodal head that fuses these cues to generate complete masks. A three-stage transfer learning strategy enhances generalization: pretrain on the excised pod synthetic dataset, transfer to the on-branch synthetic dataset, and fine-tune with a small set of real on-branch images.

The phenotypic parameter extraction module computes geometric and structural traits (focusing on SPP counting) using complete masks. The pipeline follows pose normalization → axial geometric modeling → multi-scale fused density mapping → peak detection and watershed segmentation → topological-consistency refinement, improving counting stability under occlusion/adhesion while outputting pod length, width, and area.

Through these modules, synthetic data supplies low-cost labels, amodal segmentation reconstructs complete masks, and phenotypic extraction derives accurate traits—enabling efficient, precise, and automated on-branch pod trait acquisition.

### 3.1. Synthetic Dataset Module

As indicated in Figure 1a, the synthetic dataset module consists of the excised pod overlap simulation and the on-branch mounting simulation. Its goal is to create high-quality, controllably occluded training data at low cost, with dual-mask labels (visible + amodal) for each instance—addressing real image scarcity, annotation difficulty, and complex occlusions. In this setting, synthesis is used as a task-aligned source to enrich occlusion patterns and paired visible–amodal supervision, and we assess its value through improvements on downstream metrics.

Compared with prior work [30] that only provides visible masks for excised pods on simple backgrounds, our module extends to on-branch scenes and adds amodal labels, better supporting complex phenotyping tasks. The core is algorithmic scene simulation: leveraging easily annotated excised pods and branch components to automatically generate datasets, avoiding the labor of annotating densely occluded real images.

- The excised pod dataset emphasizes morphological diversity, training basic pod feature extraction.
- The on-branch dataset simulates natural growth patterns (dense occlusions, structured layouts), strengthening the model’s occlusion robustness.

**Figure 2:**
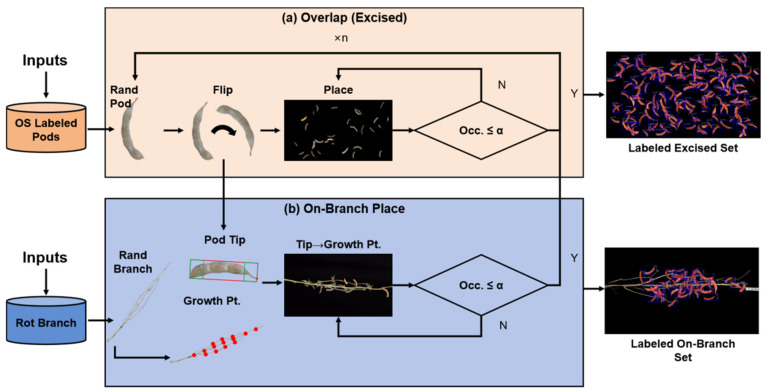
Illustration of the synthetic dataset module. (**a**) Excised pod overlap simulation: single pods are rotated/placed with controlled occlusion. (**b**) On-branch placement simulation: pods are mounted on branches per growth priors, with natural occlusion.

During synthesis, each excised pod sample has a complete mask for initial supervision. For on-branch images, the system simultaneously outputs visible masks (exposed regions) and amodal masks (full, occlusion-free shapes)—providing learning signals for amodal segmentation. The specific pipeline is illustrated in Figure 2.

#### 3.1.1. Excised Pod Overlap Simulation

An occlusion-controlled overlap algorithm is designed to explicitly manage occlusion ratios. Single-pod cutouts (transparent backgrounds) are collected to form a pod library. Using a black canvas, the algorithm samples pods, applies random in-plane rotation, and proposes placement locations—with occlusion checks before finalizing placement.

In the excised pod synthetic dataset branch, we design an overlap simulation algorithm that explicitly controls the occlusion ratio. Single-pod cutouts with transparent backgrounds are first collected from an open soybean pod dataset to form a pod library. Using a pure black canvas, the algorithm repeatedly samples a pod image from the library, applies a random in-plane rotation (to diversify pose), and proposes a random placement location.

Before accepting a placement, we evaluate whether it would cause unacceptable occlusion to any already placed pod. Let $M_{j}$ denote the complete mask of the j-th pod after geometric transformation (rotation + translation). When tentatively placing the current pod i, for every pod j already on the canvas (including i itself), we compute the region of j that becomes occluded by any other pod.

(1) $$A_{j}^{\mathrm{overlap}}=\underset{k\neq j}{⋃}\;\left(M_{j}^{\mathrm{full}}\cap M_{k}^{\mathrm{full}}\right)$$

where $M_{j}^{\mathrm{full}}$ is the index set of previously accepted pods. The occlusion ratio of pod j is

(2) $$\delta_{j}=\frac{\mathrm{Area}(A_{j}^{\mathrm{overlap}})}{\mathrm{Area}(M_{j}^{\mathrm{full}})}$$

If any $\delta_{j}$ exceeds the user-specified threshold α, the placement is rejected and a new location/angle is resampled for pod k; otherwise, the placement is accepted. After accepting, we update the visible mask for each pod as

(3) $$M_{j}^{\mathrm{vis}}=M_{j}^{\mathrm{full}}\backslash \underset{k\neq j}{⋃}\;M_{k}^{\mathrm{full}}$$

i.e., it is the complete mask minus the union of all overlapping masks from other pods.

**Figure 3:**
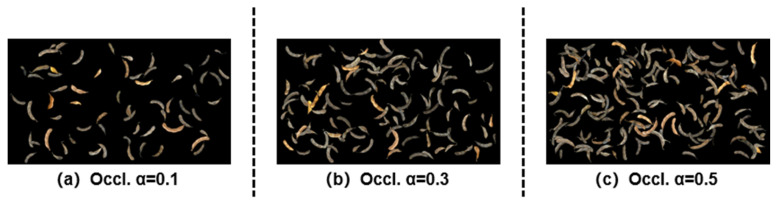
Examples of the excised pod overlap simulation. Panels (**a**–**c**) correspond to occlusion thresholds α = 0.1, 0.3, and 0.5. Higher α permits greater overlap and typically yields images with more pods.

This strategy maintains plausible but controlled occlusions—sufficient to train robustness while avoiding severe hiding of any single pod—and yields an excised pod synthetic dataset suitable for training. Figure 3 shows examples at different thresholds.

#### 3.1.2. On-Branch Placement Simulation

To emulate how pods attach to branches under natural growth, we propose an on-branch placement algorithm that yields structurally plausible synthetic images. We first collect branch images with a black background and extract the foreground branch mask via thresholding, producing transparent background branch cutouts and forming a branch library $D_{\mathrm{stem}}$.

For each branch image, we determine a feasible growth height interval $\left[h_{min},h_{max}\right]$ from the top/bottom bounds of its foreground mask. We partition the height evenly into 7 bands. On each band boundary, we add a small random vertical jitter to improve naturalness, yielding perturbed horizontal lines

(4) $$G=\overset{7}{\underset{i=1}{⋃}}\;\{(x_{i1},y_{i}),(x_{i2},y_{i}),\ldots \}$$

Each pod is aligned to one of the candidate growth points and attached to the branch in a biologically plausible orientation. To enable accurate attachment, we propose a geometry-driven tip vertex extraction method. Specifically, we fit a minimum-area rotated bounding rectangle to the pod mask and partition it, along the long axis, into four equal-width strips. We then compute the overlap between the mask and the two terminal strips; the terminal with the smaller overlap is taken as the tip side. From this side, we collect the mask boundary points intersecting the rectangle’s short edge and use their centroid as the tip vertex. Aligning this vertex with the selected growth point yields the initial placement.

To ensure reasonable inter-pod occlusions, we adopt an occlusion ratio control identical to that in the excised pod simulator. After each placement, we check whether the new pod causes any already placed pod to exceed the occlusion threshold, defined as the area of its occluded region divided by the area of its complete mask. If any pod exceeds the threshold α, the placement is rejected, and a new growth point and/or rotation is resampled.

**Figure 4:**
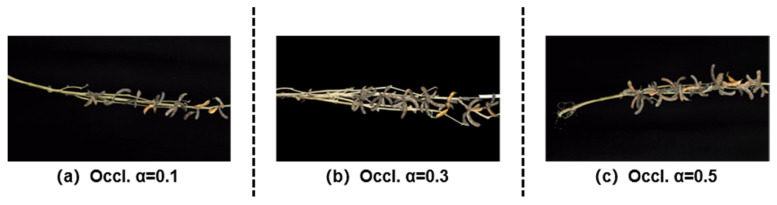
On-branch placement simulation. Panels (**a**–**c**) show composites generated with occlusion thresholds of 0.1, 0.3, and 0.5, respectively. As the threshold increases, more pods can be accommodated at a single growth point.

With these designs, the on-branch placement simulator produces composites that respect plant physiology and exhibit natural occlusion patterns, providing structurally sound training data for on-branch detection and analysis. Figure 4 shows examples at different thresholds.

### 3.2. Amodal Segmentation-Based Pod Segmentation Module

As indicated in Figure 1b, this module targets on-branch pod images with complex occlusions, outputting complete (amodal) masks for each pod—providing a basis for phenotypic extraction. Unlike conventional segmentation (visible regions only), amodal segmentation infers full shapes from visible evidence [40], reconstructing occluded regions at low annotation cost.

**Figure 5:**
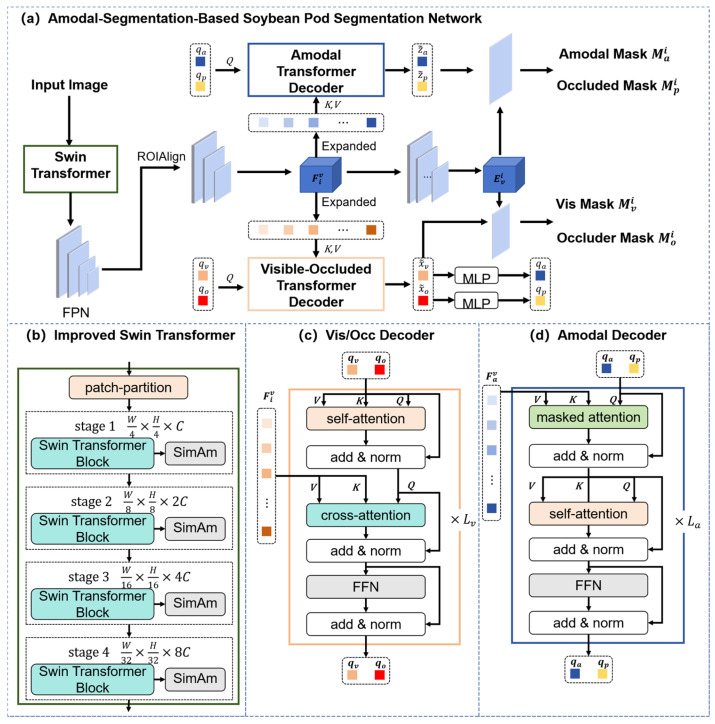
Amodal segmentation framework. (**a**) Overall structure: RoI extraction → visible/occluded heads → amodal head. (**b**) RoI extraction module: Swin Transformer Blocks + SimAM attention. (**c**) Visible mask head: Transformer decoder fuses visible/occluder embeddings. (**d**) Amodal mask head: Transformer decoder fuses amodal/occluded embeddings.

Inspired by ShapeFormer [41] (which avoids label asymmetry in bidirectional schemes), we design a pod-specific amodal network with three core components (Figure 5a) as follows:

- The RoI extraction module improves the Swin Transformer to extract multi-scale pod features.
- Visible/occluded mask heads. A Transformer decoder predicts visible/occluded region masks in parallel.
- The amodal mask head fuses above masks to reconstruct full pod morphology.

On top of the above, this head completes the occluded regions and reconstructs full pod morphology, yielding the complete (amodal) mask.

To cope with the scarcity of real on-branch occlusion data, we adopt a three-stage transfer learning schedule to exploit synthetic data and improve generalization to real scenes.

- Pretrain on the excised pod synthetic dataset to learn basic pod morphology.
- Transfer to the on-branch synthetic dataset to strengthen understanding of realistic occlusions and spatial structure.
- Fine-tune on a small real on-branch set to mitigate distribution shift.

Through the co-design of the amodal network and the transfer strategy, we achieve complete mask segmentation under occlusion, providing a reliable foundation for low-annotation-cost extraction of soybean pod phenotypic parameters.

To address incomplete masks caused by occlusion in on-branch soybean pods, we propose a Transformer-based visible-to-amodal network that infers the shapes of occluded regions from the visible evidence and ultimately outputs complete (amodal) masks. The network consists of three parts—a Region of Interest (RoI) extraction module, visible/occluded mask heads, and an amodal mask head—which, respectively, (i) extract multi-scale, salient features for pod regions, (ii) separate and predict masks for visible and occluded areas, and (iii) reconstruct the full pod structure on this basis to achieve high-quality amodal segmentation. The structure and implementation are as follows.

#### 3.2.1. RoI Extraction with an Improved Swin Transformer

To strengthen structural modeling and saliency recognition of pod regions under complex backgrounds, we design an RoI extraction module based on an improved Swin Transformer, as illustrated in Figure 5b. The module adopts a hierarchical design, stacking multiple Swin Transformer Blocks for multi-scale feature extraction. As a windowed Vision Transformer [42], Swin leverages local window self-attention and shifted-window strategies to reduce computational cost while capturing both local and long-range dependencies, yielding strong image structure modeling.

To further boost local saliency, we insert the SimAM (Simple Attention Module) [43] after each Swin Block. SimAM is a parameter-free, lightweight attention mechanism that enhances features along the channel dimension via neuron-importance scoring, significantly improving responses on key regions—particularly for small objects in complex scenes—without additional training. Its formulation is given as follows:

(5) $$s_{i}=\frac{(x_{i}-\mu {)}^{2}}{\sigma^{2}+\epsilon }$$

where $x_{i}$ denotes the activation of a neuron at a spatial location within a channel, μ and σ are the channel-wise mean and standard deviation, and ϵ is a small constant to avoid division by zero. Being parameter-free and training-free, SimAM directly amplifies responses over salient regions.

The module is organized into multiple stages, each composed of several Swin Blocks followed by SimAM, achieving spatial compaction while preserving semantic continuity. Patch Merging performs downsampling between stages, guiding the transition from local perception to higher-level semantic representation. Finally, the module outputs multi-scale feature maps rich in contextual semantics and salient structural cues; after RoIAlign, the target region features are extracted for the subsequent visible/occluded recognition and amodal completion heads.

#### 3.2.2. Transformer Decoder-Based Visible/Occluded Mask Heads

To simultaneously identify the visible regions of a pod and the regions occluded by other pods or branches, we design Transformer decoder heads for the two masks (see Figure 5b). RoI features from the previous module are fed into this head. We initialize two learnable mask queries, one for the visible part and one for the occluded part. In parallel, we extract attention features with three conv layers and obtain a semantic feature map via a transpose-conv followed by a 1×1 conv.

A dedicated visible/occluded Transformer decoder—stacked self-attention and cross-attention layers—decodes the two queries, capturing the contextual dependencies between visible and occluded areas. The decoder outputs a visible embedding $E_{i}^{v}$ and an occluded embedding $F_{i}^{v}$.

First, the RoI extraction module outputs a feature map $F_{i}$, which is fed into this head. We initialize two learnable mask queries to encode the semantics of the visible and occluded regions, respectively. Meanwhile, we extract attention features $F_{y}$ with three convolutional layers and obtain a semantic feature map $E_{y}$ by a transposed convolution followed by a 1×1 convolution; this map is used for subsequent mask prediction.

Next, the designed visible/occluded Transformer decoder decodes the two queries through stacked self-attention and cross-attention layers, thereby modeling the contextual relations between visible and occluded areas. The resulting visible embedding $\lambda_{v}$ and occlusion embedding $\lambda_{o}$ are then combined with the semantic feature map $E_{y}$ via pointwise multiplication to generate the visible mask $M_{i}^{v}$ and the occlusion mask $M_{i}^{o}$ as follows:

(6) $$M_{v}^{i}=\sigma ({\tilde{x}}_{v}⊙E_{i}^{v}),M_{o}^{i}=\sigma ({\tilde{x}}_{o}⊙E_{i}^{v})$$

where ⊙ denotes element-wise multiplication and σ(⋅) is the sigmoid.

To train this head, we adopt the binary cross-entropy (BCE) loss to supervise the visible mask $M_{v}^{i}$ and the occlusion mask $M_{o}^{i}$. BCE measures the discrepancy between predicted probabilities and ground-truth labels, and it is defined as

(7) BCE(p,y)=−[y⋅log(p)+(1−y)⋅log(1−p)]

where p is the model prediction (here, the per-pixel mask probability) and y is the corresponding ground-truth label (0 for background, 1 for target region). The losses for the two masks are

(8) $$L_{\mathrm{vis}}=\frac{1}{N}\sum_{j=1}^{N}\;\mathrm{BCE}(M_{v}^{i}[j],G_{v}^{i}[j])$$

(9) $$L_{\mathrm{occ}}=\frac{1}{N}\sum_{j=1}^{N}\;\mathrm{BCE}(M_{o}^{i}[j],G_{o}^{i}[j])$$

where N is the number of pixels in the mask and $G_{v}^{i}$ and $G_{o}^{i}$ are the ground-truth masks for the visible and occluded regions, respectively. This loss offers stable gradients and fast convergence, and is used to optimize the discrimination accuracy of the predicted masks.

#### 3.2.3. Amodal Mask Head Based on a Transformer Decoder

To reconstruct the complete structure of an occluded soybean pod, we design an amodal mask head built on a Transformer decoder (see Figure 5c).

The inputs to this module are the visible embedding ${\tilde{x}}_{v}$ and the occlusion embedding ${\tilde{x}}_{o}$ from the previous head. We project them with multi-layer perceptrons (MLPs) into an amodal semantic space, obtaining the amodal mask query $q_{a}$ and the occluded-region mask query $q_{p}$. Then, from the input RoI features $F_{i}$, we extract attention features $F_{i}^{a}$ and a semantic feature map $E_{i}^{a}$ for mask generation.

The amodal Transformer decoder takes $q_{a}$, $q_{p}$, and $F_{i}$ as inputs. Through stacked self-attention and cross-attention layers, it models the semantic–structural relations between the complete shape and the occluded region and outputs an amodal embedding ${\tilde{z}}_{a}$ and an occluded-region embedding ${\tilde{z}}_{p}$. These are combined with the semantic feature map $E_{i}^{a}$ via pointwise multiplication to produce the complete mask $M_{a}^{i}$ and the occluded-region mask $M_{p}^{i}$ as follows:

(10) $$M_{a}^{i}=\sigma ({\tilde{z}}_{a}⊙E_{i}^{a}),M_{p}^{i}=\sigma ({\tilde{z}}_{p}⊙E_{i}^{a})$$

where ⊙ denotes element-wise multiplication and σ(⋅) is the sigmoid.

To train this head, we again use binary cross-entropy (BCE) to supervise the complete mask and the occluded-region mask. The losses are defined as

(11) $$L_{\mathrm{full}}=\frac{1}{N}\sum_{j=1}^{N}\;BCE(M_{a}^{i}[j],G_{a}^{i}[j])$$

(12) $$L_{\mathrm{part}}=\frac{1}{N}\sum_{j=1}^{N}\;\mathrm{BCE}(M_{p}^{i}[j],G_{p}^{i}[j])$$

where $G_{a}^{i}$ is the ground-truth complete pod mask, $G_{p}^{i}$ is the ground-truth occluded region mask, and N is the number of pixels in the mask. With these losses, the model enforces global consistency of the complete structure while refining local visible/occluded semantics, thereby improving amodal accuracy and robustness to complex occlusions.

The overall loss is

(13) $$L_{\mathrm{total}}=L_{\det}+L_{\mathrm{cls}}+L_{\mathrm{vis}}+L_{\mathrm{occ}}+L_{\mathrm{full}}+L_{\mathrm{part}}$$

where $L_{det}$ and $L_{\mathrm{cls}}$ denote the detection and classification losses; $L_{\mathrm{vis}}$ and $L_{\mathrm{occ}}$ denote the visible and occluded region losses; and $L_{\mathrm{full}}$ and $L_{\mathrm{part}}$ denote the complete mask and occluded region losses, respectively (definitions in Equations (7)–(12)).

#### 3.2.4. Transfer Learning Strategy

Transfer learning is an effective deep learning paradigm that transfers knowledge learned on a source task to a target task, improving generalization and robustness while alleviating overfitting under limited data. It plays a key role in soybean pod detection.

On-branch and off-branch (excised) pod data are highly similar in color, shape, edge texture, and even occlusion patterns—especially in our synthetic datasets, which cover diverse occlusion and arrangement cases. Transferring pod morphology features and occlusion patterns learned on one dataset to another can markedly enhance recognition and segmentation across scenarios.

Although prior work has transferred from excised pod samples to on-branch samples [22], on-branch pods are concentrated near stem nodes and exhibit spatial organization distinct from off-branch pods; naive transfer can, therefore, degrade performance. To boost amodal segmentation in real, complex environments—and to cope with the scarcity and labeling cost of real on-branch data as well as the distribution gap—we adopt a staged transfer learning strategy. It guides the model gradually from basic morphology learning to complex on-branch scene modeling, improving generalization and robustness without sacrificing training efficiency. The three stages are as follows:

1. Phase I—foundation (excised pods, synthetic + real). Construct a training set from synthetic excised pod data and real excised pod images and fine-tune the improved ShapeFormer-based network (initialized with generic pretraining). The model learns fundamental pod structure and morphology, yielding reliable single-pod recognition.
2. Phase II—structure and occlusion (on-branch synthetic). Transfer the Stage I model to the on-branch synthetic dataset, which emulates realistic growth conditions, spatial layouts along branches, attachment patterns, and diverse occlusions. This stage teaches the model on-branch geometry and occlusion semantics, strengthening structural reasoning in complex scenes.
3. Phase III—realism (on-branch real, few-shot fine-tuning). Fine-tune the Stage II model on a real on-branch dataset to adapt to real-world illumination, background clutter, and unpredictable occlusions. This further improves robustness and domain generalization, yielding an amodal segmenter suitable for real laboratory images.

This three-stage schedule, driven by data characteristics and task complexity, progressively fuses synthetic and real data. It not only mitigates the performance drop of direct transfer but also significantly improves the accuracy and robustness of amodal segmentation in real scenes, providing a stable foundation for downstream SPP counting and precise computation of pod length/width/area.

### 3.3. SPP Extraction Module

As indicated by (c) in Figure 1, building on the proposed amodal segmentation model, we obtain complete masks of soybean pods under complex on-branch occlusions—i.e., shapes that include both visible and occluded parts. This capability overcomes the limitation of relying solely on visible masks, which miss contour information, and it thus provides complete support for downstream geometric and structural phenotyping, in particular enabling automated and accurate computation of the SPP trait.

SPP not only directly reflects per-plant yield potential but also serves as an important basis for evaluating varietal genetic characteristics. Traditional counting relies on manually excising pods; although accurate, it is time-consuming, labor-intensive, and destructive to in situ structure, making it unsuitable for large-scale, high-throughput field phenotyping. Existing automated approaches typically depend on visible masks; in on-branch scenes where occlusion is pervasive, they often suffer from missed detections and erroneous merges, leading to a marked drop in counting accuracy.

To address these issues, we propose an SPP counting method guided by the amodal segmentation results. The core workflow is “pose normalization → axial geometric modeling → multi-scale fused density mapping → peak detection and watershed segmentation → topological-consistency optimization.” The method fully exploits the morphological information provided by complete masks, balancing local particle detection with global structural constraints, and significantly improves counting stability and accuracy under heavy occlusion.

**Figure 6:**
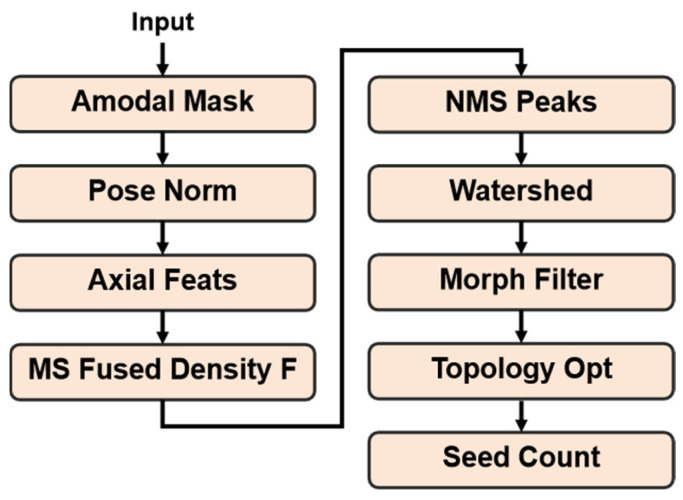
Flowchart of the SPP counting method based on amodal masks.

The overall pipeline is illustrated in Figure 6, and from top to bottom, it includes mask input, geometric modeling, density map construction, seed-center detection and segmentation, morphological refinement, and final counting output.

#### 3.3.1. Pose Normalization and Mask Preprocessing

On-branch soybean pods in images may appear at different rotation angles and poses. If seed-center detection is performed directly, inconsistencies in the long-axis direction lead to irregular center alignment, increasing false/missed detections and interfering with subsequent axial geometric computations. To ensure geometric consistency, we first extract the largest connected component from the binarized mask and fit a minimum-area rotated bounding rectangle to obtain the principal axis angle as follows:

(14) $$\theta =\arg\;\underset{\theta }{\min}\;Area(RotatedBBox(M,\theta ))$$

Using a 2D affine transform, the mask is rotated to make the principal axis horizontal and cropped to the minimal bounding box, yielding a standardized mask. To suppress segmentation noise, small components with an area of <$A_{min}$ are removed. This step reduces projection error in center localization, shrinks the search space, and improves the efficiency of later feature computations.

#### 3.3.2. Axial Width and Multi-Scale Fused Density Mapping

A single-scale distance transform is prone to failure under seed size variation and boundary gaps caused by occlusion. Since on-branch pods can exhibit substantial size variation during growth and occlusions often break contours, we require a density representation robust to both effects.

1. Axial geometric feature extraction.

On the standardized mask $M_{rot}$, compute the Euclidean distance transform D(x,y), whose value is the shortest distance from a pixel to the mask boundary. For each column x, take the column maximum and multiply by 2 to obtain the local width as follows:

(15) $$w(x)=2\cdot \underset{y\in M_{\mathrm{rot}}}{\max}D(x,y)$$

Use the median of all nonzero column widths as the standard width $\bar{w}$ and define the standard radius

(16) $$r=\kappa \cdot \bar{w}$$

where κ=0.4 is an empirical factor.

2. Multi-scale fused density construction.

Relying on a single-scale distance map can fail under pronounced size changes or boundary loss. We, therefore, fuse multi-scale Gaussian smoothing with boundary cues to construct a composite density map as follows:

(17) $$F=\alpha N_{D}+\beta N_{M}+\gamma N_{E}$$

where $N_{D}$ is the normalized distance field; $N_{M}$ is the maximum multi-scale response; and $N_{E}$ is the inverted boundary gradient map. Weights are set to α=0.45,β=0.40,γ=0.15.

This fused map FFF forms strong responses at seed centers while maintaining robustness to seed size variation and boundary incompleteness.

#### 3.3.3. Peak Detection and Marker-Controlled Watershed Segmentation

In densely packed or adhered seed rows, purely peak-based detection tends to merge neighboring seeds, whereas forcing splits can lead to over-segmentation. We, therefore, detect peaks and then perform spatially constrained segmentation guided by those peaks.

1. Non-Maximum Suppression (NMS).

We first apply NMS to obtain an initial set of seed-center candidates $P_{0}$. Each peak must be the strongest response within a disk of radius $d_{min}$ and also exceed an absolute threshold $\tau_{abs}$ as follows:

(18) $$P_{0}=\left\{p_{i}\backslash middle|F(p_{i})=\underset{q\in N\left(p_{i},d_{\min}\right)}{\max}F(q),F(p_{i})\geq \tau_{abs}\right\}$$

where $d_{min}=\rho \cdot r$, with ρ=0.8 being the radius factor and $\tau_{abs}=0.15$ being the intensity floor.

The resulting $P_{0}$ aligns well with true centers in most cases, but peak merging can still occur when inter-seed spacing is smaller than $d_{min}$.

2. Marker-controlled watershed.

To resolve adhesion, we adopt a marker-controlled watershed. The set $P_{0}$ serves as foreground markers, and high-gradient bands near the mask boundary provide background markers; flooding on the guidance surface then yields the partition.

Let G=‖∇X‖ be the gradient magnitude (Sobel) of the standardized mask region, let $M_{f}$ be the foreground markers, and let $M_{b}$ be the background markers. The watershed objective is

(19) $$\underset{S}{\min}\sum_{\left(x,y\right)\in S}\nabla F\left(x,y\right)s.t.S⊃M_{f},\;S\cap M_{b}=\emptyset$$

This constrained minimum-cut formulation guarantees that each foreground marker seeds a unique and complete region, thereby separating adhered seeds in space.

3. Post-processing filter.

For all watershed regions $S_{j}$, we apply a second-stage selection using geometric/morphological criteria as follows:

(20) $$A_{min}\leq \left|S_{j}\right|\leq A_{max},e_{j}\leq e_{max}$$

where $A_{min}=\pi (r_{min}{)}^{2}$ and $A_{max}=\pi (r_{max}{)}^{2}$ bind the minimum/maximum seed area, and $e_{j}$ is the region eccentricity with $e_{max}=0.98$ that is used to reject thin elongated artifacts.

**Figure 7:**
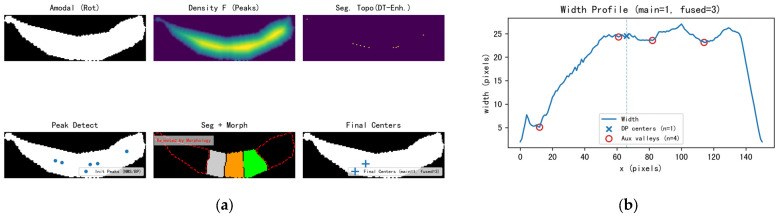
(**a**) Intermediate results: pose-normalized amodal mask, fused density F for peak detection, segmentation topography, initial peaks (blue dots), Seg + Morph (kept regions; red dashed contours mark regions rejected by morphological criteria), and final centers. In final centers, “+” marks denote DP-selected centers (the main centers), and the legend reports both the main and the fused count. (**b**) Width profile w(x) with DP centers (×) and auxiliary valleys (○). The title reports main and fused, explaining potential differences in challenging occlusions.

In Figure 7a (Seg + Morph), regions rejected by these criteria are contoured with red dashed lines and labeled Rejected by Morphology. The surviving peak set $P_{c}$ is then passed to the subsequent topological refinement step.

#### 3.3.4. Topological Consistency Optimization and Count Output

Even after watershed segmentation, complex occlusions can still cause missed or spurious detections; relying only on local cues may lead to an unstable global count. Therefore, we perform a global optimization that exploits the spatial regularity of seed distribution along the pod’s long axis.

To enhance layer-wise stability of the final count, we introduce a dynamic programming (DP)-based topological consistency selection.

1. Axial ordering and spacing model.

First, sort the candidate seed centers $P_{c}$ by their x-coordinates along the long axis as follows:

(21) $$A_{min}\leq \left|S_{j}\right|\leq A_{max},e_{j}\leq e_{max}$$

Compute the neighbor spacings $\delta_{i}=x_{i+1}-x_{i}$ and estimate the theoretical mean spacing from the local mean of the width profile w(x) as follows:

(22) $$\bar{\delta }=\eta \cdot \frac{1}{n}\sum_{i=1}^{n}w(x_{i})$$

where η=1.0 is a scale factor.

2. DP objective.

Define the score of each candidate center as follows:

(23) $$S_{i}=s_{i}-\lambda_{1}(\delta_{i}-\bar{\delta }{)}^{2}-\lambda_{2}o_{i}$$

where $s_{i}=F(p_{i})$ is the fused-density response at the candidate, $(\delta_{i}-\bar{\delta }{)}^{2}$ penalizes spacing deviation with $\lambda_{1}=0.8$, and $o_{i}$ is the overlap ratio between the candidate’s region and its neighboring regions, with $\lambda_{2}=0.6$.

The DP recursion is

(24) $$cost[i]=\underset{j<i}{\max}\left(cost[j]+S_{i}\right)$$

(25) $$prev[i]=\arg\underset{j<i}{\max}\left(cost[j]+S_{i}\right)$$

where $S_{i}$ is the per-candidate score defined in Equation (22). We sort candidates along the pod axis, hence j<i in Equations (23) and (24). cost[i] denotes the maximum total score of any valid subset that ends at candidate i; prev[i] is the index of the predecessor of i that attains cost[i] (−1 if i starts the sequence). Backtracking over prev[⋅] yields the optimal ordered subset of centers; the cardinality of this subset is taken as the main count $C_{main}$.

In addition, we derive an auxiliary count $C_{aux}$ from the axial width profile w(x) by detecting valleys in 1−w(x). The final SPP is obtained by fusing the DP count and the profile-based count with a rule that prefers the profile evidence when it is consistent and sufficiently confident and otherwise keeps the DP estimate.

This optimization strategy preserves a stable global count even when occlusions are present or local detections fail.

By chaining pose normalization, axial geometric modeling, multi-scale density analysis, marker-controlled watershed segmentation, and dynamic programming-based topological refinement, the module establishes a pipeline from complete (amodal) masks to stable counting. It effectively overcomes the missed detections and erroneous splits that plague visible mask methods under heavy occlusion, achieving accurate counting in complex scenes.

To illustrate each stage, Figure 7 presents the intermediate results from amodal mask input to the final count, including pose normalization, density mapping, segmentation topography, initial peak detection, segmentation with morphological filtering, and final center extraction.

Built on amodal masks with strong structural recovery, the proposed phenotypic-parameter module enables automatic SPP estimation under complex occlusions. It also naturally extends to extract geometric traits—pod length, pod width, and pod area—providing comprehensive phenotype information for breeding decisions and yield-component modeling.

## 4. Results

In this section, we conduct a comprehensive evaluation of the proposed amodal segmentation-based method for acquiring soybean pod phenotypic parameters. We first define evaluation metrics to verify the method’s effectiveness. Section 4.1 describes the datasets. Section 4.2 details the metrics used to assess segmentation performance, phenotypic parameter accuracy, and the utility of synthetic data. Section 4.3 presents the experimental setup and the results with analysis, including environment, training details, and baselines, ablations of each module, and comparisons with state-of-the-art methods.

### 4.1. Datasets

To validate our method, we use both real and synthetic datasets. The real data come from an off-branch (excised) soybean pod dataset [30], an on-branch public dataset [33], and an in-house soybean branch dataset. For the on-branch public data, we re-annotated the imagery so that labels include not only visible masks but also amodal (complete) masks, better serving the amodal segmentation task.

#### 4.1.1. Public Dataset of Excised Soybean Pods

This dataset originates from Yang et al. [30] on high-throughput pod phenotyping, designed for dense, excised-background instance segmentation and trait measurement. Original images were captured with an iPhone 8 Plus under strictly controlled conditions (black velvet background, fixed height 30 cm, no extra lighting) at a native resolution of 3024 × 4032 pixels. They cover eight cultivars (e.g., BJ103, BJ125), each with five plants (40 raw images in total). Pods were manually excised and randomly spread to simulate dense contact.

#### 4.1.2. Public On-Branch Soybean Pod Dataset

This dataset is integrated from Xiang et al.’s YOLO POD work [33], focusing on the detection and counting of pods attached to plants in the field. It contains three subsets (Chongzhou, Renshou2021, Renshou2022) with 2243 high-resolution RGB images (resolutions from 3960 × 2392 to 5184 × 2916). Images were collected in field trials in Sichuan, China, using DSLR cameras (Canon 700D/750D) or industrial cameras (Hikvision) under natural light, with a light-absorbing black cloth backdrop; camera height was 120–150 cm to cover the entire plants. Original annotations provided bounding boxes only.

For amodal segmentation—especially to recover complete shapes under occlusion—we upgraded the labels. On top of boxes, we provide fine instance masks and, crucially, amodal masks that outline the complete pod contour, even when parts are occluded by leaves/neighboring pods, greatly increasing the dataset’s value for learning full pod structures in occluded scenes.

#### 4.1.3. In-House Soybean Branch Dataset

Collected by our lab using an iPhone 14, soybean branches were suspended and rotated at a fixed speed during video capture; frames were extracted at 1 fps. We obtained 421 images from different viewing angles at a resolution of 1080 × 1920 pixels [44].

To further expand data scale and improve generalization, we employ the synthetic-data module (excised pod overlap simulation and on-branch mounting simulation) to generate synthetic datasets based on the real data. Both synthetic sets include amodal mask labels.

#### 4.1.4. Excised Pod Synthetic Dataset

Generated from the public excised pod dataset. We simulate random placement and controlled occlusion on a plain background to create dense-contact scenarios. Each image has a resolution of 1920 × 1080; we generate 2000 images. Every sample contains multiple pod instances with two mask types, a visible mask and an amodal mask of the complete structure, supporting phenotypic extraction under occlusion.

#### 4.1.5. On-Branch Synthetic Dataset

This dataset is constructed from the public excised pod dataset and our in-house branch images. By simulating natural attachment along branches and realistic occlusion relationships, we produce images close to real growth conditions. The resolution is 1920 × 1080; we generate 2000 images. Each image is paired with amodal masks covering complete pod contours, suitable for occlusion-rich on-branch segmentation and phenotypic analysis.

### 4.2. Evaluation Metrics

To assess the segmentation quality of the visible masks and amodal masks, we adopt the COCO-style mask AP metric and report AP50 and AP75 as key indicators. Definitions are as follows.

For a predicted mask M and a ground truth mask G, the intersection over union is

(26) $$IoU(M,G)=\frac{\left|M\cap G\right|}{\left|M\cup G\right|}$$

At a fixed threshold τ∈[0,1], we sort all predictions by confidence (high → low) and perform one-to-one matching (each ground truth instance can be matched to at most one prediction). Matches with IoU≥τ are counted as true positives (TPs); the rest are false positives (FPs). Varying the confidence threshold yields a precision–recall curve P(R). The average precision at τ is the area under this curve

(27) $$AP@\tau =\int_{0}^{1}P(R)\;dR$$

computed numerically via discrete interpolation. We report

AP50=AP@0.50,AP75=AP@0.75

AP50 is more tolerant to boundary deviations and reflects overall matching and instance separation, and AP75 is stricter and more sensitive to boundary accuracy and shape recovery under occlusion, and it is thus better at indicating high-quality amodal reconstruction.

To evaluate the accuracy of phenotypic parameters extracted from complete masks, we choose three common regression metrics tailored to practical breeding needs: the mean absolute error (MAE), the root mean square error (RMSE), and the coefficient of determination $R^{2}$. They measure agreement between predicted traits (e.g., pod length/width, seeds per pod) and manual measurements as follows:

(28) $$MAE=\frac{1}{N}\sum_{i=1}^{N}|pred_{i}-gt_{i}|$$

(29) $$RMSE=\sqrt{\frac{1}{N}\sum_{i=1}^{N}(pred_{i}-gt_{i}{)}^{2}}$$

(30) $$R^{2}=1-\frac{\sum_{i=1}^{N}(pred_{i}-gt_{i}{)}^{2}}{\sum_{i=1}^{N}(gt_{i}-\bar{gt}{)}^{2}}$$

Here, $pred_{i}$ is the predicted value for the i pod (e.g., length, width, or seed count), $gt_{i}$ is the corresponding manual measurement, $\bar{gt}$ is the mean of ground truths, and N is the number of samples. The MAE reflects average deviation; the RMSE penalizes large errors more heavily, emphasizing consistency/stability; and $R^{2}$ quantifies explanatory power with values closer to 1, indicating a better fit.

Considering the high cost of manually annotating complete masks, we further validate the effectiveness of synthetic data and transfer learning via ablations that compare three training regimes: (i) real only, (ii) synthetic only, and (iii) synthetic pretraining + few-shot real fine-tuning. Performance differences in both segmentation and phenotypic prediction indirectly demonstrate how synthetic labels can replace difficult amodal annotations to reduce labeling cost. To mitigate potential subjectivity/noise in manual labels, we also provide qualitative comparisons and quantitative analyses between predicted visible/amodal/occluded masks and ground truth, together with consistency checks for the automatically extracted phenotypic traits.

In sum, the chosen metrics jointly target segmentation accuracy, complete-shape recovery, and trait extraction quality, enabling a multi-level, comprehensive, and objective evaluation of our method.

### 4.3. Comparative Experiments

To thoroughly validate the proposed amodal segmentation-based pipeline under complex on-branch scenes, we conduct a systematic evaluation from multiple perspectives: instance segmentation accuracy under occlusion, ability to model amodal structure, and accuracy of phenotypic measurements. We compare our method against representative amodal approaches, AISFormer [45] and ShapeFormer [41], as well as mainstream instance segmentation baselines, Mask R-CNN [27] and SOLOv2 [35]. We also perform ablations to quantify the contribution of key architectural components. In addition, to assess the practical utility of amodal masks for trait estimation, we compute pod length, width, and SPP via classical image processing routines and compare them with ground truth to reveal how mask quality supports downstream tasks.

#### 4.3.1. Experimental Environment

All experiments are run on a local workstation with an Intel Core i7-14700K CPU, 64 GB of RAM, and an NVIDIA RTX 4090 (24 GB) under Ubuntu 22.04 LTS. Models and the training code were implemented in PyTorch 2.5.1 [46] compiled with CUDA Toolkit 12.4 [47] and cuDNN 8.9.7, and the codebase uses Python 3.12 [48]. Primary libraries used for data processing and analysis include NumPy 2.2.6 [49] and Matplotlib 3.10.0 [50].

To better fit the occlusion-rich mask task, the amodal network is trained with a three-stage transfer learning schedule as follows:

- Stage 1: train on the excised pod synthetic dataset to learn basic morphological structure;
- Stage 2: transfer to the on-branch synthetic dataset to learn complex occlusions and background context;
- Stage 3: fine-tune on ~400 real annotated images to adapt to real-world statistics.

Training hyperparameters for all models include an initial learning rate of 0.0005, an Adam optimizer, 100 epochs, and a batch size of 2. The loss is a weighted combination of mask BCE loss and boundary IoU loss.

#### 4.3.2. Segmentation Algorithm Comparison

**Figure 8:**
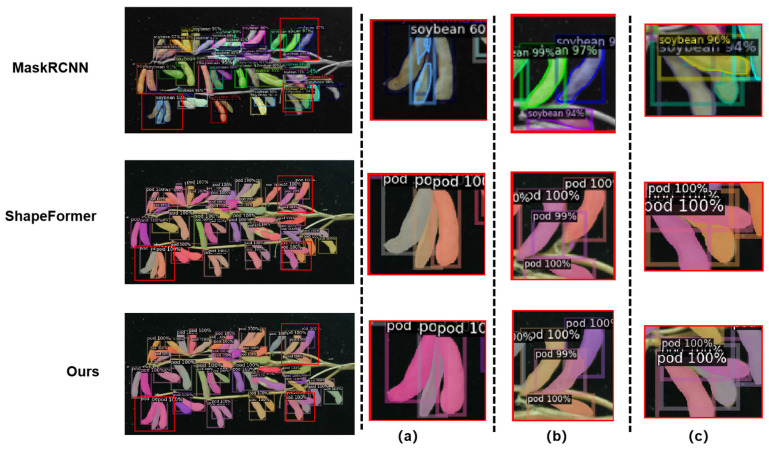
Visualization of pod mask predictions under occlusion: (**a**) light overlap, (**b**) partial occlusion, (**c**) severe occlusion.

For amodal mask prediction, our method and all four baselines are trained and tested under the same annotation protocol, with two training regimes used to assess the value of synthetic data: real only (trained solely on real data) and synthetic + real (trained on real data plus synthetic data). To make the effect of occlusion explicit, Figure 8 organizes three representative on-branch cases: (a) light overlap, (b) partial occlusion, and (c) severe occlusion. Traditional baselines without an amodal head (Mask R-CNN, SOLOv2) can only segment the visible parts and often show boundary breaks or adhesions under occlusion, failing to recover complete pod masks. Shape-centric approaches (AISFormer, ShapeFormer) better capture global shape, yet they still produce misconnected contours or ambiguous inter-pod boundaries when parts are hidden. In the specific examples of Figure 8, under light overlap (a), Mask R-CNN tends to merge neighboring pods and leak across boundaries; under partial occlusion (b), our method correctly separates the targets and recovers the occluded portion, yielding continuous visible boundaries and a complete amodal mask; and under severe occlusion (c), our method preserves clear inter-pod boundaries and shows finer edge delineation than ShapeFormer in the zoomed views, producing more faithful complete-shape recovery for downstream geometry and SPP estimation.

Quantitatively (COCO metrics; Table 1), with real-data only training, our method reaches AP50 = 87.8 on visible masks, outperforming Mask R-CNN (85.2) and SOLOv2 (86.7); for amodal masks, it attains AP50 = 84.2, exceeding ShapeFormer (77.8) and AISFormer (80.6).

With synthetic + real training, all models improve; our method rises to AP50 = 90.1 and AP75 = 74.7 on amodal masks, clearly ahead of ShapeFormer (84.5/69.5) and AISFormer (85.4/70.3). Under extreme occlusion, our approach maintains a stable high AP75 (66.3 → 74.7), whereas ShapeFormer fluctuates more (60.2 → 66.5), indicating stronger occlusion reasoning and generalization robustness.

**Figure 9:**
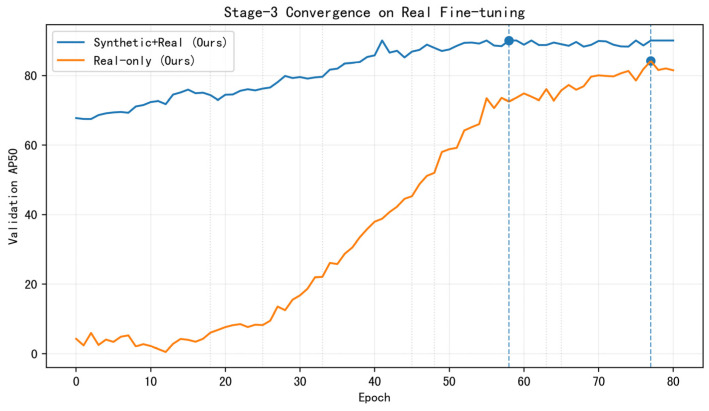
Stage 3 convergence on the real on-branch validation set. Real only starts near zero and converges to 84.2; synthetic+real starts high and reaches 90.1. Vertical dashed lines mark the best epochs for each regime. Both curves are evaluated on the same validation split as Table 1.

Convergence on real fine-tuning involves the following. To complement the above comparisons, Figure 9 reports Stage 3 convergence on the real on-branch validation set. Real only begins near zero and gradually reaches 84.2, whereas synthetic+real warm-starts at ≈64 AP50 and converges to 90.1. The markedly different curve shapes indicate that synthetic pretraining provides a strong occlusion-aware initialization, accelerates convergence, and improves the final optimum under complex overlaps.

Moreover, switching from real to synthetic + real training yields consistent gains for all models. Classical instance methods improve their visible-mask AP50 by ~+4.0 points on average (e.g., Mask R-CNN 85.2 → 89.2), while amodal methods improve amodal mask AP50 by ~+5.4 points on average (e.g., AISFormer 80.6 → 85.4). This shows that synthetic data not only enlarges training samples with complete (amodal) masks but also compensates for deficiencies in occlusion types in real data—especially under heavy occlusion and dense cross-over—thereby enhancing learning of occlusion recovery strategies. Overall, our method achieves the best performance across metrics, validating the effectiveness of the amodal design and synthetic-to-real transfer for soybean pod phenotyping.

#### 4.3.3. Phenotypic Parameter Computation and Validation

To evaluate practical utility in agricultural phenotyping, we compute seeds per pod on the test split of the public excised pod dataset. The pipeline is pose normalization → axial geometric modeling → multi-scale fused density → peak detection + marker-controlled watershed → topological-consistency optimization, fully exploiting the complete morphology provided by amodal masks. To quantify the benefit of occlusion completion, we run the extractor with visible and amodal masks as inputs; we primarily report the amodal mask results using the MAE, RMSE, and $R^{2}$ (Section 3.1).

With amodal support, we also obtain additional geometric traits automatically.

- Pod length. Take the long side of the minimum-area rotated rectangle along the principal axis.
- Pod width. Compute the maximum inscribed circle radius from the Euclidean distance transform twice.

All three metrics reach high accuracy: SPP achieves an MAE = 0.07 seeds and an RMSE = 0.26 seeds; pod length and pod width obtain an MAE = 2.87 px and 3.18 px, respectively. By completing key geometry in occluded regions, amodal masks reduce systematic bias in length/width/count and improve overall consistency and robustness.

**Figure 10:**
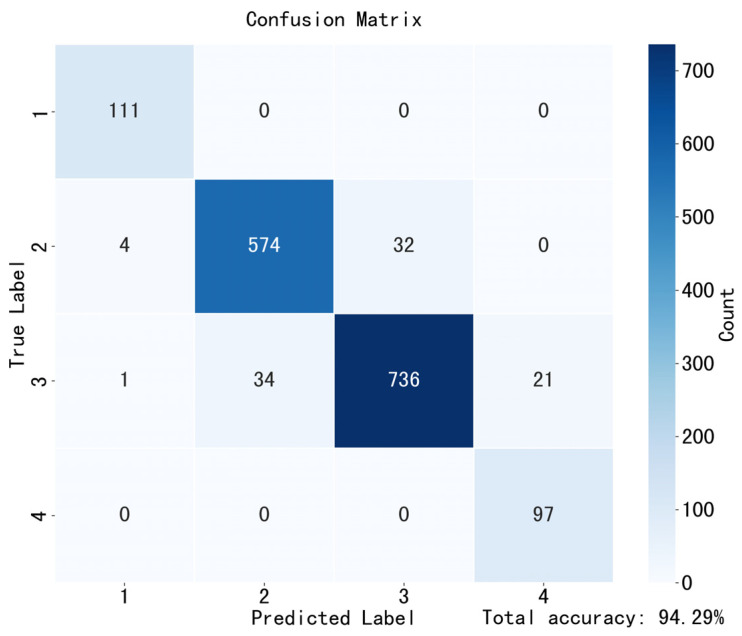
Confusion matrix of SPP prediction (1–4 seeds) on the excised pod test set. The x-axis is the predicted label; the y-axis is the ground-truth label. Darker color indicates more samples. The strong diagonal and adjacent-class errors (2–3, 3–4, 1–2) confirm high agreement.

Further, discretizing SPP into 1–4 classes, the confusion matrix in Figure 10 shows an overall accuracy of 93.3%; all errors occur between adjacent classes (±1 seed), hence Acc@±1 = 100%, which is consistent with the low MAE/RMSE in Table 2.

#### 4.3.4. Ablation Study of the Amodal Segmentation Network

To investigate how the backbone and attention modules affect performance, we evaluate three configurations. A baseline with ResNet-50 was used as the feature extractor, (1) replacing the backbone with a Swin Transformer to strengthen long-range dependency modeling and (2) inserting the SimAM module to enhance structural modeling over salient regions. All three models are trained under the synthetic + real regime and evaluated on the common test set.

To isolate the effects of the backbone and the attention module, we adopt a stepwise ablation with three configurations: (1) baseline—ResNet-50 as the feature extractor; (2) Swin—replace ResNet-50 with a Swin Transformer to better capture long-range dependencies and multi-scale context; and (3) Swin+SimAM—on top of (2), insert SimAM after each Swin stage to enhance structure-aware responses in salient regions. All models are trained under the same synthetic+real training regime and evaluated on a common test set with identical optimization and augmentation settings.

As shown in Table 3, upgrading the baseline from ResNet-50 to Swin Transformer yields clear gains. Visible AP50 rises from 86.7 → 90.5 (+3.8) and AP75 from 70.1 → 75.1 (+5.0); amodal AP50 improves 84.5 → 88.3 (+3.8) and AP75 69.5 → 72.4 (+2.9). These results confirm that the Transformer’s global modeling substantially improves boundary quality.

Adding SimAM delivers the best overall performance: visible AP50 91.6 (+1.1 over Swin) and AP75 77.6 (+2.5); amodal AP50 90.1 (+1.8) and AP75 74.7 (+2.3). This indicates that SimAM notably facilitates fine-structure recovery in occluded regions; its lightweight attention effectively sharpens focus on key structural areas while simultaneously improving visible mask segmentation, validating the soundness and practicality of our design.

## 5. Discussion

To address the challenges of complex occlusion, structural clutter, and the high labeling cost of amodal (complete) masks for on-branch pods in laboratory settings, we propose a soybean pod phenotyping pipeline that couples synthetic data generation with amodal segmentation. The pipeline combines (i) a dual-mask synthetic dataset, (ii) an improved Swin+SimAM backbone for amodal recovery, and (iii) a morphology-driven trait module. Experiments show that, while reducing labeling cost, the method markedly improves structural restoration accuracy and the reliability of trait extraction. On lab on-branch samples, the approach performs strongly and provides a dependable technical basis for fast, standardized, high-precision acquisition of yield-related traits in smart breeding.

Our study explicitly targets lab on-branch samples. Our goal is not in-field, in situ dynamic monitoring but to supply high-precision, standardized organ-level geometric baselines (length/width/seeds) for breeding. Controlled conditions (standard background, fixed illumination, and distance) ensure imaging consistency and repeatability, enabling quantitative comparisons for variety screening, genetic analysis, and yield component assessment. Field phenotyping and lab phenotyping are complementary and equally important in the breeding pipeline; the latter offers high-SNR “baseline measurements,” while the former captures spatiotemporal dynamics at the population scale. On complex, occluded lab samples, our method recovers complete masks and delivers stable counting, substantially boosting both throughput and accuracy and offering direct practical value.

For amodal mask prediction, our method shows comprehensive advantages. On the real on-branch test set, our method consistently outperforms baselines across standard IoU-based metrics; qualitative examples show fewer mask breaks and more continuous boundaries under occlusion. Together, the three-stage transfer strategy (progressive domain adaptation) and the improved Swin+SimAM design jointly enhance boundary continuity and complete-shape integrity under occlusion. These segmentation gains translate directly into phenotypic accuracy; length/width errors remain low across datasets, and SPP errors are small and stable. The confusion matrix (Figure 10) indicates high overall accuracy, with errors confined to adjacent classes (±1 seed), indicating stable recovery of seed topology from amodal masks.

In sum, our method effectively restores occluded pod structure and extracts key phenotypic parameters, directly serving the demand for high-precision phenotypes in soybean assessment and modern breeding. It is important to stress that our current optimization and validation target lab on-branch data; this setting aims to provide precise baseline traits for breeding, which is different from but complementary to field phenotyping oriented to in situ, dynamic monitoring for crop management. Direct deployment to real, open, dynamic field scenarios—recovering accurate traits under natural growth—still faces tougher challenges (background noise, varying illumination, severe occlusion, complex growth patterns). Remaining challenges concern scale calibration across devices and robust handling of extreme occlusions and atypical morphology.

### Limitations and Future Work

1. Scale calibration and transfer

Although synthetic data lowers labeling cost, converting pixels to physical units still requires manual calibration, and the lack of unified standards forces repeated procedures across devices/environments. We will investigate multi-view imaging and depth sensor fusion for automatic calibration and develop an adaptive scale-mapping model to remove manual intervention and improve absolute accuracy and cross-scenario consistency.

2. Extreme occlusion and atypical morphology

Under extreme occlusion or deformed shapes, predicted amodal boundaries can still be uncertain. A root cause is that static synthetic data cannot fully emulate complex natural variation, limiting generalization to atypical forms. Future directions include GAN-based dynamic growth modeling to increase biological realism of synthetic data and 3D reconstruction with spatial constraints to strengthen reasoning about hidden structures and fundamentally improve boundary prediction.

## 6. Conclusions

We present a synthetic-to-real approach for amodal pod segmentation and phenotypic extraction in lab on-branch scenes. On the segmentation side, the method achieves strong performance on standard visible and amodal metrics; on the trait side, it yields precise length/width estimates and stable SPP counting. While reducing labeling cost, the framework produces reproducible, high-quality phenotype data that directly support variety selection and genetic analysis.

The core ideas—amodal supervision via dual-mask synthesis, transfer learning across synthetic and real domains, and morphology-aware counting under complete-shape constraints—are general and transferable. Beyond soybean pods, the approach can extend to organ-level phenotyping tasks, such as wheat spikes and maize ears. In future work, we will pursue systematic adaptation and validation in more challenging in-field settings to provide a general, reliable computer vision solution for precision agriculture and digital breeding.

## Figures and Tables

**Table 1:** Visible vs. amodal segmentation on the on-branch soybean test set.

Method	Training Data	Visible Mask Seg		Amodal Mask Seg	
		AP50 (%) ↑	AP75 (%) ↑	AP50 (%) ↑	AP75 (%) ↑
Mask R-CNN	Real Only	85.2	60.8	-	-
	Synth + Real	89.2	74.3	-	-
SOLOv2	Real Only	86.7	62.5	-	-
	Synth + Real	90.3	75.1	-	-
AISFormer	Real Only	76.8	62.2	80.6	63.2
	Synth + Real	81.9	66.5	85.4	70.3
ShapeFormer	Real Only	77.5	63.4	77.8	60.2
	Synth + Real	86.7	70.1	84.5	69.5
Ours	Real Only	87.8	68.5	84.2	66.3
	Synth + Real	91.6	77.6	**90.1**	**74.7**

↑ indicates higher is better. Bold and underline indicates the best result.

**Table 2:** A summary of errors against manual measurements.

Phenotypic Parameters	MAE ↓	RMSE ↓	R^2^ ↑
Seeds per Pod	0.07	0.26	0.87
Pod Length (px)	2.87	5.2	1
Pod Width (px)	3.18	6.1	0.94

↑ indicates higher is better. ↓ indicates lower is better.

**Table 3:** Performance of different amodal segmentation configurations (synthetic + real).

Model Architecture	Visible Mask Seg		Amodal Mask Seg	
	AP50 (%) ↑	AP75 (%) ↑	AP50 (%) ↑	AP75 (%) ↑
ResNet-50 (Baseline)	86.7	70.1	84.5	69.5
+ Swin Transformer	90.5	75.1	88.3	72.4
+ SimAM (Proposed Method)	**91.6**	**77.6**	**90.1**	**74.7**

↑ indicates higher is better. Bold and underline indicates the best result.

## Data Availability

The data presented in this study are available upon request from the corresponding author.
